# Need for improved access to HIV prevention programmes

**Published:** 2010-12

**Authors:** Trevor A. Hart

**Affiliations:** Director, HIV Prevention Lab, Department of Psychology, Ryerson University and the HIV Social, Behavioural & Epidemiological Studies Unit, Dalla Lana School of Public Health, University of Toronto, Canada

HIV prevalence continues to grow worldwide. The United Nations Programme on AIDS (UNAIDS) estimates that there are 33.4 million people living with HIV, a total that is more than 20 per cent higher than in 2000^1^. India accounts for half of Asia’s HIV prevalence, with approximately 2,270,000 persons living with HIV in India in 2008. Like many Asian countries, India has a concentrated epidemic, in which three high risk groups, female sex workers (FSWs), men who have sex with men (MSM), and injection drug users (IDUs) have HIV prevalences that are 10 - 20 times more than the prevalence found among women presenting at antenatal clinics. Migrant workers and truckers are also viewed at higher risk, as potential bridge populations between these high risk groups and the general population^2^.

Much of the increase in HIV prevalence worldwide is due to the increased life expectancy among people with HIV/AIDS who are receiving antiretroviral therapies^3^. Although we are far from providing universal access to treatment for people with HIV/AIDS, this progress is remarkable. In addition to providing treatment, scaling-up HIV prevention services may substantially decrease the costs of treatment by lowering the number of people who need HIV treatment^4^. There is, therefore, still a pressing need for an expansion and improvement in HIV prevention services.

In India, there have clearly been some successes in HIV prevention, as 0.45 per cent of the adult population was infected with HIV in 2002, as opposed to 0.29 per cent in 2008^2^. This decline may be due to decreasing incidence. For example, among women aged 15-24 yr who were tested nationally at antenatal clinics, HIV prevalence declined by 54 per cent between 2000 and 2007 in the southern States of Andhra Pradesh, Karnataka, Maharashtra, and Tamil Nadu^5^. HIV incidence may also be decreasing in some areas among high risk groups, such as female sex workers in Karnataka^6^.

Much of the reduction in HIV incidence in India may be due to the combined governmental efforts of the National AIDS Control Organization (NACO), civil society, and non-governmental organizations, such as the Avahan India AIDS Initiative, to reach as many members of high risk groups as possible with HIV prevention services. Much of these prevention services have included needle/syringe exchange for injection drug users, provision of free condoms for high risk groups, and sexually transmitted infection (STI) referral and treatment. There is also a programme to improve the safety of blood transfusions across all populations in India^2^. NACO has also expanded HIV information and behaviour change programmes, with extensive use of mass media to promote condom use. For persons living with HIV, NACO is promoting better access to antiretroviral medications, including for prevention of mother-to-child transmission of HIV, and management and prevention of opportunistic infections^2^.

There are conflicting data as to the success of this ambitious HIV prevention agenda. Mathematical modeling shows that to achieve population level effects on reducing HIV incidence and prevalence, at least 60 per cent of the specific population must be reached^7^. Using this criterion, clearly some advances have been made. For example, NACO and the Avahan India AIDS Initiative report providing almost universal coverage for HIV prevention services for men who have sex with men in Andhra Pradesh, Karnataka and Maharashtra^8^. However, there are difficulties in knowing the exact number that would designate universal coverage, especially given that the three high risk groups (female sex workers, injection drug users, and men who have sex with men) may be reluctant to self-identify due to fears of being stigmatized. In addition, some data suggest insufficient coverage even using the estimates available for high risk groups. Among IDUs, there are 1.3 needle and syringe exchange programmes per 1000 injection drug users, an amount which may reach only 61.4 per cent of IDUs, in India^9^. Regardless of the precise numbers needed to calculate whether universal access to HIV prevention services is being achieved, more HIV prevention services are clearly needed as long as new infections occur.

Recent research reveals several biomedical and psychosocial methods that may help to further reduce HIV infection in India and worldwide. Among biomedical prevention methods, expanding access to antiretrovirals may also have some effect in reducing HIV incidence. A recent meta-analysis suggests that the HIV transmission rate from a person with HIV on antiretroviral medication is approximately 0.5 per 100 person-years, as opposed to is 5.6 per 100 person-years for persons with HIV not on antiretroviral medication^10^. Mathematical modelling studies have also suggested that improving access to HIV testing and counselling could reduce HIV infection rates^11^,^12^. Vaccines may also play an important role in the future, although research is still early in development to be of service to HIV prevention providers at the present time.

With 88.6 per cent of infections in India due to unprotected sexual intercourse, universal access to HIV prevention services is needed for people at risk through heterosexual or same-sex sexual activity. However, each of these methods has significant limitations that may prevent universal coverage. For example, although male circumcision has been endorsed as a preventative measure by the World Health Organization and UNAIDS^13^, the history of male circumcision in India as primarily a religious practice could create a barrier in its implementation among non-Muslim populations. Vaginal microbicides are being tested as a method to protect women from HIV. One of these, a tenofovir gel showed a 39 per cent reduction in new HIV infections^14^. However, other data suggest that microbicides may be less acceptable among Indian women who have a primary sexual partner, because of fears that its use would imply that the woman has been unfaithful or mistrusted her partner^15^. Pre- and post-exposure prophylaxis using antiretrovirals are also to be considered, although the acceptability of taking antiretrovirals for non-HIV infected people may not be high^16^.

Until there is a biomedical HIV prevention method that is 100 per cent effective and acceptable at a population level, behavioural methods are critical to HIV prevention efforts. In addition, partial protection from a biomedical method will likely not be effective in reducing HIV incidence if people at risk for or living with HIV reduce their self-imposed constraints on engaging in high risk behaviour, and therefore, increase their high risk behaviours^16^,^17^. Among behavioural methods, NACO’s promotion of condom use acceptability^2^is an important step in the right direction, but there is also a need for research-based HIV prevention interventions that have efficacy and effectiveness data in India, especially among high risk groups. In one such approach, the United States Centers for Disease Control and Prevention has supported the implementation of empirically-supported HIV prevention interventions nationwide at community-based organizations and local health agencies^18^. Although the effectiveness of this policy on HIV incidence remains to be seen, this is an innovative way to link HIV prevention science to HIV prevention services. Given the cultural differences between India and the Western and African countries in which many interventions have been evaluated by research, India may benefit from its own co-ordinated effort to identify behavioural HIV prevention interventions that are efficacious and effective in Indian high risk groups. These interventions may be adapted from those from other countries or created in India to match the cultural values in India. With India’s diversity in culture, language, religion, and in the effects of HIV across and within States, interventions should also be adapted specifically to match the local context.

HIV prevention interventions must also include structural interventions, such as reducing stigma against high risk groups that may make members of these groups less likely to identify themselves for HIV surveillance and less likely to access HIV prevention services. Structural interventions are beneficial because of their direct effects on promoting human rights of HIV affected and infected communities. These interventions include India’s repealing of section 377 of the penal code, which had banned homosexuality. By decriminalizing homosexuality, India has increased the chances of men who have sex with men to be able to mobilize their communities to participate in epidemiologic surveillance, to get appropriate access to HIV treatment, and to even more actively participate in HIV prevention initiatives. Improving and increasing access to needle and syringe exchange programmes is also important in order to allow access for injection drug users to health care and HIV prevention services. Lastly, the stigma of living with HIV needs to be reduced if we hope to provide universal access to HIV treatment and prevention.

In summary, a multifaceted approach incorporating biomedical, behavioural, and structural interventions is crucial if we are to have universal access to HIV prevention. Although some countries, including India, have made substantial progress on this front, it will be important not only to provide services but also to measure the effect of each of these services in reducing HIV among high risk groups and in the general population. HIV prevention must remain a top priority if we hope to finally reduce the HIV pandemic worldwide.
